# Low Molecular Weight Heparin in Portal Vein Thrombosis of Cirrhotic Patients: Only Therapeutic Purposes?

**DOI:** 10.1155/2014/895839

**Published:** 2014-12-29

**Authors:** Raffaele Licinio, Mariabeatrice Principi, Giuseppe Losurdo, Nicola Maurizio Castellaneta, Enzo Ierardi, Alfredo Di Leo

**Affiliations:** Gastroenterology Section, Gastroenterology Unit, Department of Emergency and Organ Transplantation, University of Bari, Azienda Universitario-Ospedaliera Policlinico, Piazza G. Cesare 11, 70124 Bari, Italy

## Abstract

Cirrhosis has always been regarded as hemorrhagic coagulopathy caused by the reduction in the hepatic synthesis of procoagulant proteins. However, with the progression of liver disease, the cirrhotic patient undergoes a high rate of thrombotic phenomena in the portal venous system. Although the progression of liver failure produces a reduction in the synthesis of anticoagulant molecules, a test able to detect the patients with hemostatic balance shifting towards hypercoagulability has not yet been elaborated. The need of treatment and/or prophylaxis of cirrhotic patients is demonstrated by the increased mortality, the risk of bleeding from esophageal varices, and the mortality of liver transplantation, when portal vein thrombosis (PVT) occurs even if current guidelines do not give indications about PVT treatment in cirrhosis. In view of the general feeling that the majority of cirrhotic patients at an advanced stage may be in a procoagulant condition (suggested by the sharp increase in the prevalence of PVT), it is presumable that a prophylaxis of this population could be of benefit. The safety and the efficacy of prophylaxis and treatment with enoxaparin in patients with cirrhosis demonstrated by a single paper suggest this option only in controlled trials and, currently, there are no sufficient evidences for a recommendation in the clinical practice.

## 1. Introduction

Coagulation alterations are associated with liver cirrhosis and often they are evident when the disorder is complicated by hemorrhagic or thrombotic events as well as when a surgical operation is required. However, the topic is often underestimated in current literature. On these bases, we believe that a review of the main aspects regarding liver cirrhosis and coagulation disorder is of interest, including pathophysiological features and related therapeutic problems.

### 1.1. Laboratory and Clinical Pictures

Cirrhosis has always been regarded as an example of disease characterized by alterations of conventional coagulation tests, especially the prothrombin time (PT-INR), caused by the reduction in the hepatic synthesis of procoagulant proteins (factors II, VII, IX, X, V, and XI) [1].

However, the relationship of this aspect with hemorrhagic events such as the bleeding from esophageal varices has not been demonstrated. Indeed, there are some evidences that, after the onset of esophageal varices, the risk factors for bleeding essentially consist of haemodynamic and mechanical factors (portal pressure gradient, size of varices, and red and blue signs of varicose veins) [2].

On the other hand, with the progression of liver disease, the cirrhotic patient has a high rate of thrombotic phenomena in the portal venous system: the prevalence of these events is 1% in patients with compensated cirrhosis and up to 25% of patients on the list for a liver transplant. Moreover, in 70% of replaced livers, portal vein thrombosis (PVT) was found [3].

The risk of thromboembolic events, such as deep vein thrombosis and pulmonary embolism, has been focused on in many studies despite prolonged PT-INR and thrombocytopenia in cirrhotic patients [4, 5].

Indeed, the progression of liver failure produces a reduction in the synthesis not only of procoagulant proteins but also of anticoagulant molecules (protein C, antithrombin) while the production of factor VIII is unaffected or increased (being factor VIII secreted by endothelial cells of kidney tubules and glomeruli) [6, 7].

Further, in the advanced stages of cirrhosis, hepatic folate storage is reduced and its metabolic activation by hepatocytes is suppressed, thus causing a secondary hyperhomocysteinemia with a further imbalance towards a procoagulant condition [8].

Therefore, it is presumable that, in some patients, the hemostatic balance can be in equilibrium, but, with liver disease progression, it hangs on one side and this is not characterized by the bleeding. This balance is, moreover, extremely dynamic, since it can be reversed probably within a few hours as a result of events such as infections and acute uremia, especially in favor of a procoagulant state [9].

### 1.2. Cirrhosis and Hypercoagulopathy: Innate or Acquired?

Some authors have assumed that since not all liver diseases are intended to progress towards an evolved condition, cirrhotic patients could represent a selected population of individuals with an innate hypercoagulability.

In fact, the main data of literature suggest that an innate prothrombotic state is not frequent in cirrhotic patients, but it should be investigated when PVT arises. Indeed, it has been discovered that the G20210A mutation of the prothrombin gene is the most frequent innate alteration in cirrhotic patients with PVT [10].

It has been also demonstrated that patients with hepatitis C and another coagulation alteration (factor V Leiden) have an increased risk (more than thrice) of a rapid cirrhosis progression [11].

Consequently, it is not possible to exclude a strong association between a prothrombotic state and the rapid progression to cirrhosis in both animal models and clinical trials [12].

What is reported above may be presumed by considering that many molecular pathways are in common between inflammation, fibrosis, and coagulation and, therefore, a hypercoagulability reflects the predisposition to a most intense inflammation. For example, thrombin and factor Xa are able to activate protease activated receptors (PAR) of hepatic stellate cells [13, 14].

### 1.3. Inadequacy of Current Tests

It seems that both clinical and experimental studies confirm the presence of a procoagulant rather than hemorrhagic coagulopathy in liver cirrhosis despite the fact that clotting tests (PT-INR) appear to show an opposite picture.

In order to explain this apparent contradiction, it is important to premise that PT and PTT are based on the rate of conversion of fibrinogen to fibrin, and they precisely evaluate the fibrin that is formed as soon as only the first 5% of thrombin is produced, thus lacking the estimation of the effect of the remaining 95% of thrombin. Thus, PT and PTT evaluate only the first stage of the formation of thrombin. However, the most important limitation of these tests is that they do not take into account the thrombomodulin, which plays a key role in activating the endogenous anticoagulant system (activated protein C, APC), which is reduced in cirrhotic patients compared to healthy subjects. So, the reduction of natural anticoagulants (APC) should be considered in the balance of coagulation. Indeed, the PT is much more influenced by proclotting factors than by anticoagulant ones and consequently in cirrhotic patients although both systems are decreased, only an apparent reduction of procoagulant factors is noticeable with the current tests. Finally it has been demonstrated that, by adding the thrombomodulin to the system, the plasma of cirrhotic patients produces a greater amount of thrombin compared to controls [15]. Since PT-INR measures only the activity of procoagulants and fails to capture changes in anticoagulants, it is therefore not surprising that the PT does not predict the bleeding risk [16]. For example, PT-INR lacks the correlation of bleeding risk and therefore, in the setting of portal hypertension, its correction may paradoxically exacerbate the risk of bleeding when portal pressure is increased [17]. Castañeda et al., for instance, have demonstrated, in a mouse cirrhosis model, that the administration of procoagulant factors worsens hypovolemic shock following the bleeding [18].

## 2. Cirrhosis and Portal Vein Thrombosis (PVT)

### 2.1. Beyond Hypercoagulopathy

The procoagulant state is the first step towards the critical complication of portal vein thrombosis (PVT). The onset of this condition is dependent on two more events, that is, venous stasis and hemodynamic alterations of the vascular endothelium in order to fulfill Virchow's triad. The condition of cirrhosis is distinguished by alterations of the parenchyma (sinusoidal capillarization, contractility of stellate cells, and fibrous septa with formation of regenerative nodules) that determine portal hypertension and, therefore, a hemodynamic slowing of the flow as evidenced by Zocco et al. [19] that when under 15 cm/sec is an important predictor of PVT. On the other hand, an endothelial dysfunction characterized by increased adhesiveness of platelets has been demonstrated for cirrhosis and is directly proportional to the severity of liver disease [20].

### 2.2. Need of PVT Treatment

Although in most cases it has a clinically asymptomatic course, PVT can be a trigger for liver failure in the form of “acute” ascites nonresponsive to diuretics or bleeding varices and this makes its treatment relevant even when a severe clinical manifestation is absent. In fact, it has been estimated that the risk of bleeding from esophageal varices increases 80–120 times when PVT occurs in cirrhosis [21, 22]. Moreover, PVT is also an independent predictor of the failure of therapeutic procedures used in order to control bleeding from varices [23], and, in addition, it negatively impacts on mortality by increasing it at 3 years [24]. Therefore, the patients who are on a liver transplant list complain of the most serious consequences. In these cases PVT may preclude liver transplantation (OLT) in many liver transplant centers or may make it more difficult. PVT indeed increases the complexity of the surgery, the rate of postoperative complications (a rise of 30% has been estimated), and perioperative mortality [25]. Although it may be argued [26] that the rate of spontaneous improvement of PVT is high (47% in the cited study), this statement is valid only when PVT arises in a clinically subacute/asymptomatic way for the simultaneous development of collateral circulation. On the other hand, the thrombotic obstruction of portal system may lead to a dramatic splanchnic derangement that could compromise the life of the patient himself. In these cases, anticoagulation is compulsory. Indeed, current guidelines [27] clearly state that the goal of treatment of acute PVT is to recanalize the obstructed veins with the aim of preventing intestinal infarction and portal hypertension, and anticoagulation therapy is of proven benefit in patients with acute PVT [28]. Moreover, since PVT could represent a contraindication to liver transplantation, a prophylaxis of this event in a small subset of cirrhotic patients, that is, those on a waiting list, could be of benefit in order to reduce the mortality during this period of time.

### 2.3. Indications to Treatment

Even though rare cases of spontaneous resolution of PVT have been described [29], treatment with anticoagulants is definitely the most effective way to achieve recanalization of the portal vein, thereby improving the prognosis [30]. Unfortunately, current guidelines do not give clear indications about the type and timing of PVT treatment in cirrhotic patients despite the fact that the effectiveness of an immediate and aggressive therapy with anticoagulants for at least 6 months is proven to be effective in this condition in subjects without liver diseases [31].

Indeed, in cirrhotic patients, PVT often has a subacute or chronic clinical course for the presence of portal cavernoma. In these conditions, the indication to treat PVT is supported essentially by some studies that have shown a benefit where a congenital prothrombotic state was demonstrated (Jak-2 mutation or factor V Leiden). Anyway, it is important to emphasize that, currently, major evidences for a benefit of anticoagulation in PVT are coming from studies conducted on acute/subacute PVT [32]. Conversely, data about anticoagulation in chronic PVT, that is, portal cavernoma, are scanty and are reported on small sample sizes. Only a single case report has demonstrated the efficacy of anticoagulant drugs in portal cavernoma [33].

Another important subset of patients in which anticoagulation for PVT may be useful is constituted by patients on the waiting list for liver transplantation with PVT. In these subjects, indeed, the treatment allowed increased survival rates two years after transplantation in the subjects in which portal vein recanalization had been achieved [25]. The main anticoagulant therapy in this subset of patients is represented by a low molecular weight heparin (LMWH), as demonstrated by the studies reported in Table 1, where the different modalities of PVT therapy are illustrated [31, 34–36].

LMWHs, which were first developed in the 1980s, are produced by chemical or enzymatic depolymerisation of heparin [37]. Similarly to heparin, LMWHs bind to and inhibit a pentasaccharide sequence of antithrombin; through successive binding to clotting factors such as thrombin and factor Xa, it catalyses their inactivation by antithrombin. Differently from classical unfractioned heparin, LMWHs have an enhanced selectivity for factor Xa [38]. LMWHs have several advantages, such as the lack of needing laboratory monitoring, subcutaneous administration, and low rates of idiosyncratic thrombocytopenia. On the other hand, disadvantages are present, even if they may be less severe when compared to unfractionated heparins: LMWHs must be avoided in renal insufficiency and are less effective in dissolving clot-bound coagulation factors [39]. Overall, LMWHs have gained increasing success and are commonly employed in several diseases.

### 2.4. Prophylaxis

Prophylaxis with a LMWH, that is, enoxaparin, in cirrhotic patients with hypercoagulopathy has been proposed as an important medical facility by Villa et al. [40] in their recent single-center survey. This was a nonblinded study including 70 outpatients (18–75 years old) with cirrhosis (Child B7-C10) of any etiology without decompensation for at least 3 months and no evidence of portal thrombosis. Exclusion criteria were platelets <10.000/mm^3^ and F2 or F3 with red spots varices not subjected to elastic ligatures. Patients were randomized in two groups; the experimental arm received enoxaparin 4000 UI sc/day for 12 months and the control one did not receive any treatment. After 12 months both groups continued followup after treatment withdrawal.

The safety of prophylaxis with enoxaparin in patients with cirrhosis was demonstrated. Moreover, enoxaparin was effective in preventing PVT in cirrhotic patients within 12 months of treatment and 12 months following its withdrawal. Such prophylaxis also showed a significant reduction of liver decompensation only in the period of active treatment. All this implies a higher probability of overall survival for the patients treated with enoxaparin.

The prophylaxis of liver major complications obtained with the administration of enoxaparin could be explained by its ability to antagonize Xa factor (and so even to reduce thrombin), which can activate stellate cells, responsible for collagen deposition, that cooperate in the mechanisms of liver fibrosis. The mechanisms of wound repair and liver fibrosis share many common pathways. Therefore, it is conceivable that the model of fibrosis described in the first process could be appropriate also in hepatic development of fibrotic tissue following organ damage and lobular architecture collapse [14]. Moreover, a significant reduction of the incidence of bacterial infections (bacteremia and/or spontaneous bacterial peritonitis) was even detected in the group receiving enoxaparin. In this regard, it has been hypothesized that enoxaparin could be able to reduce intestinal permeability and bacterial translocation, thus acting on the variations of the microcirculation on the whole portal system, by preventing microthrombosis that may worsen portal hypertension in cirrhotic patients [41].

### 2.5. PVT Prophylaxis and Treatment: A Critical Analysis of Literature

Several studies involving cirrhotic patients have investigated vitamin K antagonists (VKA) or LMWH efficacy for PVT prophylaxis and treatment. Results have shown that LMWH (in particular enoxaparin) appears to be safe in patients with cirrhosis Child B and Child C with few adverse events, while, in the case of VKA, discontinuation of the drug for onset of bleeding was often needed [35, 36, 42]. Warfarin was associated with increased bleeding complications only in patients with advanced liver disease [43] and thrombocytopenia (<50.000/mm^3^) [44].

Another element making the management of VKA therapy difficult is the need of monitoring the PT-INR that in cirrhosis may not be fully reliable, thus making it difficult to deal with the correct therapeutic range.

All these considerations seem to favor LMWH for PVT prophylaxis. LMWH is easily maneuverable due to its excellent safety profile, and, in addition, it may beneficially affect the coagulation mechanisms and influence the necroinflammatory activity in the liver parenchyma [14, 45]. Nevertheless, some cautions must be considered when LMWHs are administered, as they are not risk-free. It is important to ascertain that the baseline platelet count is >10.000, because of the potential risk of idiosyncratic autoimmune thrombocytopenia. However, this cut-off has been chosen by authors of a single paper [40]; therefore it cannot be considered as a solid cut-off since a validation by other studies is required. Further, LMWHs are filtered by the kidney, so a creatinine clearance >30 mL/min is considered the ideal cut-off for a safe administration; otherwise it is compelling to monitor anti-Xa activity and make dose adjustments.

As regards the dosage for the treatment of PVT, the scheme proposed by Amitrano [35] could be the best option: 200 UI/kg/day for at least 6 months and if possible it is extended up to 17 months. For the prophylaxis 4000 IU/day for 12 months seems, to the best of our knowledge, the schedule that gave the best results [40]. However, it is important to underline that prophylactic treatment of PVT with enoxaparin has been attempted only in few studies and that, currently, there are no sufficient evidences for a recommendation of its use in the clinical practice outside controlled trials.

## 3. Future Perspectives

It seems rational to give indication to treat PVT with LMWH when the clinical presentation is either acute or chronic with cavernoma, as it is possible to reduce or eliminate the negative effects on prognosis and allow transplantation with a reduced mortality. However, this treatment has limitations due to exclusion criteria characterized by renal failure which does not allow a correct elimination of the drug, platelets <10.000/mm^3^ for the high hemorrhagic risk, and esophageal varices at high risk of bleeding unable to undergo prophylactic endoscopic treatment.

Additionally, it could be conceivable to administer enoxaparin to a subset of cirrhotic patients in order to prevent liver failure with stabilization of liver function and avoid complications when patients are listed for transplantation.

The real problem in the prophylaxis lies in the fact that a test able to detect the patients with a hemostatic balance shifted towards hypercoagulability has not yet been elaborated. In fact, while for the cirrhotic with PVT this problem does not arise because thrombosis itself is a sign of hypercoagulability, for the remaining part of the cirrhotic population it is not always clear-cut.

The general feeling is that the majority of cirrhotic patients at an advanced stage who are often included in the list for transplantation are actually in a procoagulant condition, and this is suggested by the sharp increase in the prevalence of PVT.

Thus, it is presumable that a prophylaxis of the population in an advanced state of liver disease could be of benefit and, in this regard, the patient waiting for transplantation may be a prototype. These patients could perform prophylaxis for one year and benefit from the protection from PVT for further 12 months (after prophylaxis) for total protection of 48 months, which just happens to coincide with the average waiting time for the organ in Italy [46].

Finally, enoxaparin could be largely used in the future for the prevention of PVT and liver failure in cirrhotic patients in an advanced stage of the disease when the criteria for the admission to this therapy will be well clarified and/or a diagnostic test will be pointed out to detect the population with hypercoagulopathy. Nevertheless, current evidences are not enough to define a program of prophylaxis for PVT, since studies investigating this aspect are few and are conducted on small sample sizes.

## Figures and Tables

**Table 1:** Main studies published on anticoagulation therapy for PVT in cirrhotic patients.

Reference	Number of treated patients	Treatment protocol	Success rate	Adverse events
Senzolo et al. [31]	33	LMWH 95 UI/Kg of body weight, t.i.d	36.3% complete 27.3% partial	1 nonvariceal bleeding

Delgado et al. [34]	55	LMWH in 47 pts, 21 of which were shifted to VKA later; VKA in 8 pts with target INR of 2-3	38.5% complete 12.3% partial	5 (7.7%) nonvariceal bleeding 6 (9.2%) variceal bleeding

Amitrano et al. [35]	28	Enoxaparin 200 UI/Kg/day	75% complete 7.1% partial	None

Francoz et al. [36]	19	LMWH 5700 UI/day, followed by VKA with target INR 2-3	42.1% complete	None

LMWH: low molecular weight heparin; VKA: vitamin K antagonists.
